# The gut microbiota of *Cystidicola farionis* parasitizing the swim bladder of the nosed charr morph *Salvelinus malma* complex in Lake Kronotskoe (Kamchatka, Russia)

**DOI:** 10.21307/jofnem-2021-106

**Published:** 2021-12-15

**Authors:** E.N. Kashinskaya, E.P. Simonov, P.G. Vlasenko, G.N. Markevich, A.V. Shokurova, K.B. Andree, M.M. Solovyev

**Affiliations:** 1 Institute of Systematics and Ecology of Animals of Siberian Branch of Russian Academy of Sciences; Research group of physiology and genetics of hydrobionts; Frunze St. 11, Novosibirsk, 630091, Russia; 2 University of Tyumen, Institute of Environmental and Agricultural Biology (X-BIO), 25 Lenina St., Tyumen, 625003, Russia; 3 Kronotsky State Nature Biosphere Reserve, Yelizovo; 4 Instituto de Investigación y Tecnología Agroalimentarias; Cultius Aquàtics; San Carlos de la Rapita, Tarragona, ES 08140, Spain; 5 Tomsk State University; Institute of Biology, Ecology, Soil Science, Agriculture, and Forestry; 36 Lenin Ave, Tomsk, 634050, Russia

**Keywords:** *Cystidicola farionis*, Gut microbiota, Nosed charr, *Salvelinus malma* complex, V3–V4 region of 16S rRNA, 18S and 28S rRNA

## Abstract

Using the approach of sequencing the V3–V4 region of the 16S rRNA gene, we have analyzed the bacterial diversity associated with the gut and “body” (other parts of nematode after dissection: cuticle, epidermis and longitudinal muscles, etc) of *Cystidicola farionis* parasitizing the swim bladder of different morphotypes of the nosed charr. Comparisons of the gut microbiota of nematodes with their “body” has revealed that the associated microbiota are closely related to each other. Taxonomic analysis indicated that the relative abundances of the dominant nematode-associated bacteria varied with individual fish. The common dominant microbiota of the gut and “body” of nematodes were represented by *Aeromonas*, *Pseudomonas*, *Shewanella*, and *Yersinia*, while the associated microbiota of the swim bladder of the nosed charr was dominated by *Acinetobacter*, *Cetobacterium*, *Pajaroellobacter*, *Paracoccus*, *Pseudomonas*, *Shewanella*. By comparing the associated microbiota of nematode parasitizing the different morphotypes of the nosed charr the difference in richness estimates (number of OTU’s and Chao1) were revealed between the N1g and N2 morphs.

Aquatic organisms constantly interact with different microbial communities that inhabit the surrounding environments. The microbial community is an integral and essential part of all vertebrates and invertebrates, and plays an important role in a plethora of different physiological processes including development, nutrition, reproduction, and immunity (Lathrop et al., 2011). The microbial communities are formed by bacteria and fungi, as well as archaea, viruses and protozoa (Bisen et al., 2012). Colonization of the different host tissues and organs by a microbial community leads to establishment of the symbiotic relationships between the host organism and microbiota (Glendinning et al., 2014). In the same time, in nature, the host organism has symbiotic relationships with various groups of parasites (Bisen et al., 2012).

The host-helminthes-microbiota interactions are intensively studied in mammalian systems, with emphasis given to the nematode-host interactions. The majority of these studies were conducted on the model species *Caenorhabditis elegans* in laboratory controlled cultures (Petersen et al., 2014; Zhang et al., 2017). However, the gut microbiota of free-living nematodes from natural environments has also been studied (Berg et al., 2016; Dirksen et al., 2016; Samuel et al., 2016). Experimental studies regarding the nematode-associated microbiota have shown that nematodes play an important role in host health and different physiological functions (Zheng et al., 2020). Thus, infestations by the nematode *Trichuris muris* in mice and pigs shifts their metabolism and leads to the reduction in a large number of metabolic products (Li et al., 2012; Houlden et al., 2015). In mice, the nematode infestation in the small intestine by *Heligmosomoides polygyrus* leads to an increased abundance of Lactobacillaceae and Enterobacteriaceae species (Walk et al., 2010). The health benefits to the host of probiotic use of Lactobacillaceae have been amply demonstrated in humans, other mammals and fish (Fijan, 2014), so this benefit of nematode presence, can be seen to be just as beneficial as the presence of the bacterial component of the microbiome.

Interactions between bacterial diversity found in the host and the microbes associated with the helminthes have been studied less extensively in fish. To date, it has been shown that different ecto- and endoparasites of fish are known to harbor a unique bacterial community (Hughes-Stamm et al., 1999; Poddubnaya and Izvekova, 2005; Korneva and Plotnikov, 2006; Llewellyn et al., 2017; Gonçalves et al., 2020; Kashinskaya et al., 2020, 2021). In particular, a bacterial species was found to colonize the different areas of cestode teguments of *Trianophorous nodulosus, Eubothrium rugosum* and *Ligula intestinalis* from the intestine of pike, burbot, and bream, correspondingly (Izvekova and Lapteva, 2004). Similarly, a specific bacterial community on the surface of the intestinal trematode *Gyliauchen nahaensis* was found on the dorsal surface and excretory papillae regions (Hughes-Stamm et al., 1999). Also, microbiota of the ectoparasitic *Gyrodactylus avalonia* (Monogenea) on the skin of fish was also characterized (Cusack and Cone, 1985). Several studies have also demonstrated ultrastructural features and methods of attachment of bacteria to the tegument of cestodes (Korneva and Plotnikov, 2012; Poddubnaya and Izvekova, 2005). Most of these studies reported so far are focused on the morphological and physiological characteristics of bacteria associated with parasites inhabiting the intestine of fish (Hughes-Stamm et al., 1999; Izvekova, 2003; Poddubnaya and Izvekova, 2005; Korneva and Plotnikov, 2006, 2012). There are only a few studies that have used V3–V4 16S rRNA sequencing technologies to create a metagenome library for analysis of the microbiota associated with tegument of cestodes and microbiota of ectoparasites (Llewellyn et al., 2017; Gonçalves et al., 2020; Kashinskaya et al., 2020, 2021).

Despite the ubiquity of parasitic organisms in fish, the role of the nematode-associated microbiota remains poorly understood (Gonçalves et al., 2020). Based on the relatively large body size (up to 57.0 mm according to Lankester and Smith, 1980 and our observation), the nematodes from the genus *Cystidicola* (family Cystidicolidae) can be a fit model to study fish-microbe-nematode interactions. *Cystidicola farionis* parasitizes the swim bladder of fish from the families Salmonidae and Osmeridae, with distribution in Europe, Asia, and North America (Menconi et al., 2019). *C. farionis* has a complex life cycle that includes an amphipod crustacean as first intermediate host and a fish as definitive host (Lankester and Smith, 1980; Moravec, 1994). Recent studies have demonstrated an indicative role of the abundance of *C. farionis* in trophic diversification between sympatric morphotypes of salmonids (Knudsen et al., 1996, 2006; Busarova et al., 2017). Thus, the endemic nosed charr, belonging to Salvelinus malma complex inhabiting the littoral zone of Lake Kronotskoe (Kamchatka, Russia) is divided into several morphological groups, which spawn in different grounds and can be considered as morphs (N1a, N1g, N2, and N3). The N1g, N2, and N3 morphs predominantly feed on gammarids, and are characterized by a high level of infestation with *C. farionis*; while the N1a morph feeds mostly on larvae and pupae of chironomid and on mollusks, and is characterized by a low infestation with *C. farionis* (Busarova et al., 2017).

Thus, the main aim of the present study was to estimate the composition and structure of microbial communities of the gut and “body” (cuticle, epidermis and longitudinal muscles, etc) of *Cystidicola farionis* nematodes, as well as microbiota of the swim bladder of its host – the nosed charr morphs.

## Materials and methods

### Study area and sampling

**Figure 1:**
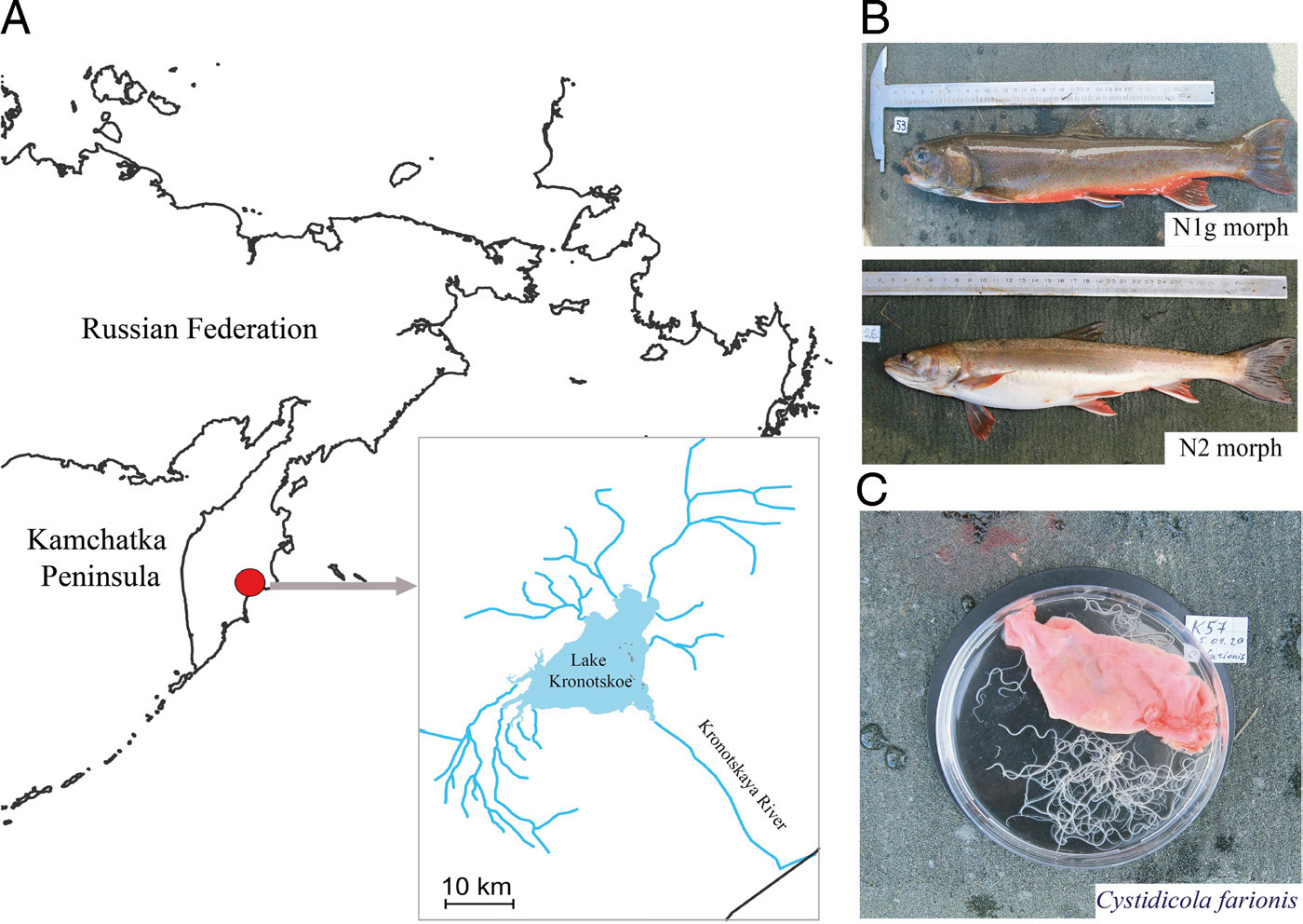
Sampling site (A) of *C. farionis* (C) parasitizing the swim bladder of different morphs of the nosed charr (B). Picture by P.G. Vlasenko.

Four individuals of the nosed charr (Fig. 1B) with total weight 370.1 ± 95.5 and total length 345.0 ± 34.2 mm were collected from September 30 to October 01, 2020 in the littoral zone of Lake Kronotskoe (Fig. 1A), which is the biggest lake in the Kamchatka Peninsula (Russia, 54°47′11″ N 160°13′36″ E). The lake area is about 246 km^2^ and has an average depth of 58 m with maximum 136 m. Many rivers and streams flow into the lake, the largest of which are the Listvennichnaya, Unana and Uzon rivers. The lake drains into the Kronotskaya River which flows 39 km southeast into the Pacific Ocean (Busarova et al., 2017). Fish were captured using gill-nets (mesh sizes 35 and 45 mm) and transported alive to the laboratory in plastic containers filled with water from the site of fish capture (duration approximately 30 min). All fish were sacrificed and aseptically dissected as previously described (Kashinskaya et al., 2015). Male and female fish were identified according to gonadal development and the type of morph (N1g or N2) was assigned (Table 1). The level of infestation by *C. farionis* in the swim bladder of analyzed fish were registered.

**Table 1.** Descriptions of samples used in this study.

Ecological group of fish	Sex	Body weight, g	Total length, mm	Fork length, mm	Type of sample for microbiome analysis	Number of pooled samples
1-N1g	F	304.4	355.0	309.0	Gut	15
					Gut	15
					“Body”	15
					“Body”	15
					Swim bladder	1
2-N1g	M	652.1	438.0	400.0	Gut	20
					“Body”	20
					Swim bladder	1
3-N2	M	229.6	292.0	260.0	Gut	5
					Gut	5
					Gut	10
					Gut	25
					“Body”	5
					“Body”	5
					“Body”	10
					“Body”	25
					Swim bladder	1
4-N2	F	294.3	295.0	333.0	Gut	20
					“Body”	20
					Swim bladder	1

### Isolation and dissection of nematodes

The swim bladder was removed aseptically from the body cavity of fish and placed into a sterile Petri dish. *C. farionis* individuals (Fig. 1C) were retrieved from the swim bladder using sterile forceps. Before dissection, the nematodes were washed three times in sterile Ringer buffer (pH = 7.4) to remove surface-adherent particles of mucus from the host swim bladder and afterward placed into a sterile Petri dish. In total, 115 individual nematodes were dissected and their guts were removed using a sterile microsurgical forceps, scissors, and scalpel. The “head” end of the nematode was fixed by forceps in order to cut it with scissors. After that, the same manipulations were repeated with the “tail” end of the nematode. Then, the gut was pulled out from the “body” by sterile forceps. All procedures were conducted under stereomicroscope with 8–64× magnification (Carl Zeiss, Stemi^TM^ DV-4). Finally, “body” remains of dissected nematodes, their guts, and mucosa from the nosed charr swim bladder were taken separately to analyze the associated microbiota. By using the term “body” hereinafter it will be understood this refers to the cuticle, epidermis and longitudinal muscles, etc. which remained after dissection of nematodes and isolation their gut. Taking into account that washing of nematodes in different solution does not mean a complete removal of bacteria from their surfaces and that muscles of nematodes have been considered to be sterile, we can then conclude that the microbiota of the “body” is mostly the microbiota of the external surfaces (cuticle) of the nematode. To check our assumptions, we used micro surgery instruments, as a more relevant method to separate the gut microbiota from microbial communities of the other nematode’s parts (cuticle, epidermis and longitudinal muscles, etc).

The samples of “body” and guts were collected in pools ranging from five to twenty individuals of nematodes (depending on their size) from each studied fish. The number of pooled biological replicates ranged from one to four for each fish although the actual number depended upon the level of infestation. (Table 1). All samples were frozen in liquid nitrogen immediately after dissection and transferred to ‒80 °C storage prior to DNA extraction.

### Parasitological analysis

During June–August of 2013, June-November of 2014, and September-November of 2020, 323 individuals of nosed charr were collected in order to estimate the prevalence and intensity of infestation by *C. farionis*. All fish were dissected and swim bladder was extracted and the number of *C. farionis* was registered. The prevalence (P), intensity, and mean abundance of parasite infestation were calculated according to the definitions by Bush et al. (1997).

The prevalence (*P*) of parasite infestation (in %) was calculated as P = I*100*/N*, where *I* is the number of infested host and *N* is the total number of hosts examined. Mean intensity of infestation (*I*) was assessed as the average of number of individuals of a particular parasite species (*K*) in a single infested host (*n*): *I = K/n*.

### Genetic identification of parasites

For genetic characterization, *Cystidicola farionis* collected from two nosed charr, were preserved in 96% ethanol and stored at 4°C until DNA extraction. Total DNA was extracted from single ethanol-preserved individuals of parasites using the DNA-sorb B kit following manufacturer’s protocols (kit for DNA extraction, Central Research Institute of Epidemiology, Russia). The 28S nuclear ribosomal large subunit rRNA gene (28S rRNA) was amplified using the following primers (forward 5′-AGCGGAGGAAAAGAAACTAA-3′, reverse 5′-TCGGAAGGAACCAGCTACTA-3′) and PCR conditions as described in Nadler et al. (2006). The fragment of the 18S nuclear ribosomal small subunit rRNA gene (18S rRNA) was amplified using the primers (forward 5′-AGCGGAGGAAAAGAAACTAA-3′, reverse 5′-TCGGAAGGAACCAGCTACTA-3′) and PCR conditions as described in Floyd et al. (2005). Double-stranded DNA was amplified using BioMaster HS-Taq PCR-Color (2x) kit (Biolabmix, Novosibirsk, Russia) according to the manufacturer’s instructions (http://biolabmix.ru/products/klassicheskaja_pcr/biomaster_hs-taq_pcr-color__2_/). The PCR products were purified by adsorption on Agencourt Ampure XP (Beckman Coulter, Indianapolis, IN, USA) columns and subjected to Sanger sequencing using the BigDye Terminator V.3.1 Cycle Sequencing Kit (Applied Biosystems, Waltham, MA, USA) with subsequent unincorporated dye removal by the Sephadex G-50 gel filtration (GE Healthcare, Chicago, IL, USA). The Sanger products were analyzed on an ABI 3130XL Genetic Analyzer (Applied Biosystems). The purification and sequencing of PCR products were performed in the SB RAS Genomics Core Facility (Novosibirsk, Russia). Manual editing, alignment of sequences, distances and phylogenetic analysis were performed with MEGA 7 (Kumar et al., 2016).

Phylogenetic reconstruction was performed using the maximum-likelihood method with the K2 + G model of nucleotide substitutions. Test of phylogeny were calculated using the Bootstrap method with 1000 replications. The nearest-match sequences from GenBank were used for phylogenetic reconstructions (Table 2). Newly obtained sequences were deposited into GenBank (NCBI) under the following accession numbers: MZ151867–MZ151869 for large subunit ribosomal RNA gene, MZ150507–MZ150509 for small subunit ribosomal RNA gene.

**Table 2.** Nearest-match identification of *C. farionis* to known sequences in NCBI database.

GenBank acc. no.	Gene	Species
JF803919.1	small subunit ribosomal RNA gene (18S) partial	*C. farionis*
MG594291.1	small subunit ribosomal RNA gene (18S) partial	*C. farionis*
DQ094172.1	small subunit ribosomal RNA gene (18S) partial	*Ascarophis arctica*
MN294781.1	small subunit ribosomal RNA gene (18S) partial	*Comephoronema werestschagini*
MN294783.1	small subunit ribosomal RNA gene (18S) partial	*Capillospirura ovotrichuria*
MG594289.1	small subunit ribosomal RNA gene (18S) partial	*Capillospirura sp.*
JF934733.1	small subunit ribosomal RNA gene (18S) partial	*Proleptus sp.*
DQ442660.1	small subunit ribosomal RNA gene (18S) partial	*Neoascarophis macrouri*
JF803921.1	small subunit ribosomal RNA gene (18S) partial	*N. longispicula*
JF803926.1	small subunit ribosomal RNA gene (18S) partial	*Heliconema longissimum*
JF803949.1	small subunit ribosomal RNA gene (18S) partial	*H. longissimum*
JF803930.1	small subunit ribosomal RNA gene (18S) partial	*Ascarophis adioryx*
MT086834.1	large subunit ribosomal RNA gene (28S) partial	*C. farionis*

### Sample preparation, DNA extraction, and 16S rDNA metagenomic sequencing of microbial communities

Before DNA extraction, parasites, “body” remains and swim bladder were collected into sterile microcentrifuge tubes with lysis buffer (300 µl) for DNA isolation and mechanically homogenized by pestle for 1 min. Following the kit manufacturer protocols, DNA was extracted using the DNA-sorb B kit (NextBio, Russia). The DNA extraction protocol was previously described in Kashinskaya et al. (2020). DNA from a sample containing only sterile deionized water was extracted and included in PCR as a negative control. Before amplification and sequencing of V3–V4 variable region of the 16S ribosomal RNA (rRNA) gene, samples with isolated DNA were mixed in equimolar concentration depending on the type of analyzed sample (Kashinskaya et al., 2015). Sequencing of the V3, V4 hypervariable regions of 16S rRNA genes was carried out on an Illumina MiSeq sequencing platform (500 cycles - 2 × 300 paired-end) by Evrogen (Moscow, Russia) using the primer pair S-D-Bact-0341-b-S-17, 5′-CCTACGGGNGGCWGCAG-3′ and S-D-Bact-0785-a-A-21, 5′-GACTACHVGGGTATCTAATCC-3′ (Klindworth et al., 2013).

The amplification conditions and other methods were applied according to the original manufacturer’s protocol (smetagenomic-libraryµl of reverse primer (1 µM), 5 µl of forward primer (1 µM) and 12.5 µl of 2× KAPA HiFi Hotstart ReadyMix (KAPA Biosystems, Wilmington, MA, USA) in a total volume of 25 µL. The PCR reaction was performed on a 96-well 0.2 ml PCR plate (Life Technologies) using the following program: 95°C for 3 min, followed by 25 cycles of 95°C for 30 s, 55°C for 30 s and 72°C for 30 s and a final extension step at 72°C for 5 min. After producing amplicons, the libraries were cleaned up and mixed in equimolecular portions using SequalPrepTM Normalization Plate Kit (ThermoFisher, Cat # A10510-01) and checked using capillary electrophoresis. Samples were multiplexed using a dual-index approach with the Nextera XT Index kit (Illumina Inc., San Diego, CA, USA) according to the manufacturer’s instructions.

### 16S rRNA gene sequence

Forward and reverse read pairs were merged and quality filtered with Mothur 1.31.2 (Schloss et al., 2009). Any reads with ambiguous sites and homopolymers of more than eight bp were removed, as well as sequences shorter that 350 or greater than 500 bp. QIIME 1.9.1 (Caporaso et al., 2010) was used for the further processing of the sequences. *De novo* (abundance based) chimera detection using USEARCH 6.1 (Edgar, 2010) was applied to identify possible chimeric sequences (‘identify_chimeric_seqs.py’ with an option ‘-m usearch61’ in QIIME). After chimera filtering, the QIIME script ‘pick_open_reference_otus.py’ with default options was used to perform open-reference OTU picking by UCLUST (Edgar, 2010), taxonomy assignment (UCLUST), sequence alignment (PyNAST 1.2.2; Caporaso et al., 2010) and tree-building (FastTree 2.1.3; Price et al., 2010). This algorithm involves several steps of both closed-reference and open-reference OTU picking followed by taxonomy assignment, where the SILVA core reference alignment (release 132; Quast et al., 2013) was used as a reference. Chloroplast, mitochondria and non-bacterial sequences were removed from further analysis. An addition, the singletons were removed from feature table.

The richness (number of OTU’s and Chao1 index) and diversity estimates (Shannon and Simpson index) per sample were calculated using QIIME 1.9.1 (Caporaso et al., 2010). Then, the samples were rarified to the lowest sequencing effort (1667 sequences) using QIIME. Such sequencing effort allowed us to include as many samples as possible in further analyses without reducing the power of the statistical methods. Increasing the sequencing effort did not change the obtained results, but reduced the number of analyzed samples. Nucleotide sequences were deposited in the Sequence Read Archive (SRA NCBI), accession number PRJNA727704.

### Statistical analyses

All data are presented as a mean ± standard error (SE). For estimating the differences between the richness and diversity estimates, a non-parametric Kruskal–Wallis test with Dunn’s multiple comparisons test was applied. A weighted UniFrac (Lozupone and Knight, 2005) dissimilarity matrix was calculated in QIIME and used for downstream analyses. The matrix was used to perform principle coordinates analysis (PCoA) to visualize differences among groups of samples. Permutational multivariate analysis of variance using distance matrices was used as implemented in the ‘adonis’ function of the vegan R package (Oksanen et al., 2018). Pairwise comparisons for all pairs of levels of used factors were performed using ‘adonis.pair’ function of the EcolUtils R package (Salazar, 2018). Analysis of multivariate homogeneity of group dispersions (variances) to test if one or more groups is more variable than the others, was performed using the ‘betadisper’ function of the vegan R-package. In all the aforementioned tests statistical significance was determined by 10 000 permutations.

## Results

### The prevalence and mean intensity of parasite infestation

Parasitological analysis of 323 individuals of nosed charr observed at different years (2013, 2014 and 2020) in Lake Kronotskoe has revealed that the prevalence of *C. farionis* ranged from 63 to 100%. The intensity of parasite infestation ranged from zero to 550 (Table 3). Prevalence levels observed in different months of 2014 were high and remained stable during all the sampling time.

**Table 3.** Prevalence, intensity and mean abundance of *C. farionis* in swim bladder of three morphs of nosed charr at different sampling times.

Year	Month	Prevalence, %	Intensity	Mean abundance	Number of fish
*Nosed charr N1 (n = 227)*^a^					
2013	VI-VIII	69	0–328	83.0 ± 3.9	13
2014	VI	96	0–452	144.0 ± 15.4	49
	VII	84	0–335	131.0 ± 13.6	50
	VIII	87	0–526	150.0 ± 18.2	38
	IX	– ^b^	–	–	–
	X	94	0–550	140.0 ± 18.2	47
	XI	100	12–508	154.9 ± 23.4	30
*Nosed charr N1g (n = 2)*					
2020	IX-X	100	15–20	16.6 ± 5.0	2
*Nosed charr N2 (n = 57)*					
2013	VI-VIII	82	0–685	172.0 ± 44.8	17
2014	VI	63	0–274	126.0 ± 42.0	8
	VII	80	0–362	179.0 ± 38.9	10
	VIII	100	43–252	118.0 ± 28.8	8
	IX	100	23	23.0	1
	X	100	130–376	243.0 ± 33.1	8
	XI	100	155–466	333.0 ± 92.0	3
2020	IX-X	100	5–45	32.5 ± 12.5	2
*Nosed charr N3 (n = 37)*					
2013	VI-VIII	88	0–519	205.0 ± 58.2	8
2014	VI	–	–	–	–
	VII	100	28–225	130.0 ± 44.5	4
	VIII	100	420	420.0	1
	IX	74	0–466	102.0 ± 24.04	23
	X	100	318	318.0	1
	XI	–	–	–	–
2020	IX-X	–	–	–	–

Notes: ^a^“a” and “g” variations of N1 morph are presented together. ^b^No data.

### Molecular identification of parasites

**Figure 2:**
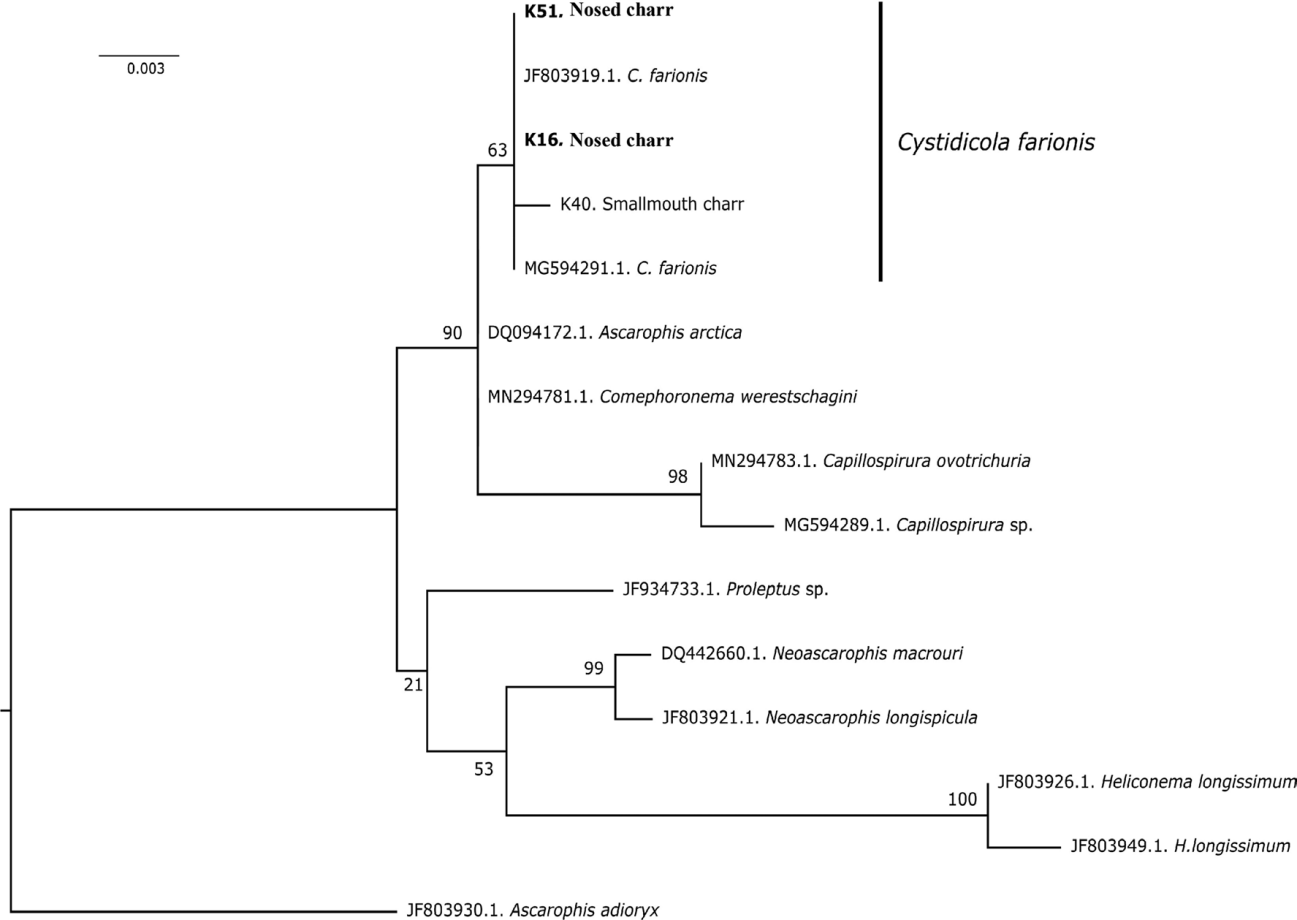
18S rRNA gene tree reconstruction based on ML methods.

Four new sequences (two 28S rRNA and two 18S rRNA sequences) were obtained from *C. farionis* parasitizing the swim bladder of nosed charr. Two sequences (28S rRNA and 18S rRNA) obtained from the *C. farionis* parasitized in the smallmouth charr were also used in the analysis. The 28S rRNA fragment with length of 866 base pairs were identical to *C. farionis* from GenBank under accession number MT086834.1. According to the 18S rRNA obtained with sequence length of 756 base pairs a phylogenetic ML-tree was constructed with *Ascarophis adioryx* as outgroup taxa (Fig. 2).

Together with the previously published sequences obtained from *C. farionis*, the original sequences formed a separate clade. The low value of bootstrap support observed for this clade was due to the low variability of the 18S rRNA gene. In general, the *C. farionis* clade was located on the tree as part of a common weakly resolved clade with *Ascarophis adioryx*, C*omephoronema werestschagini*, and the genus *Capillospirura*.

### The richness and diversity estimates of microbial communities

#### Between different sample types

**Figure 3:**
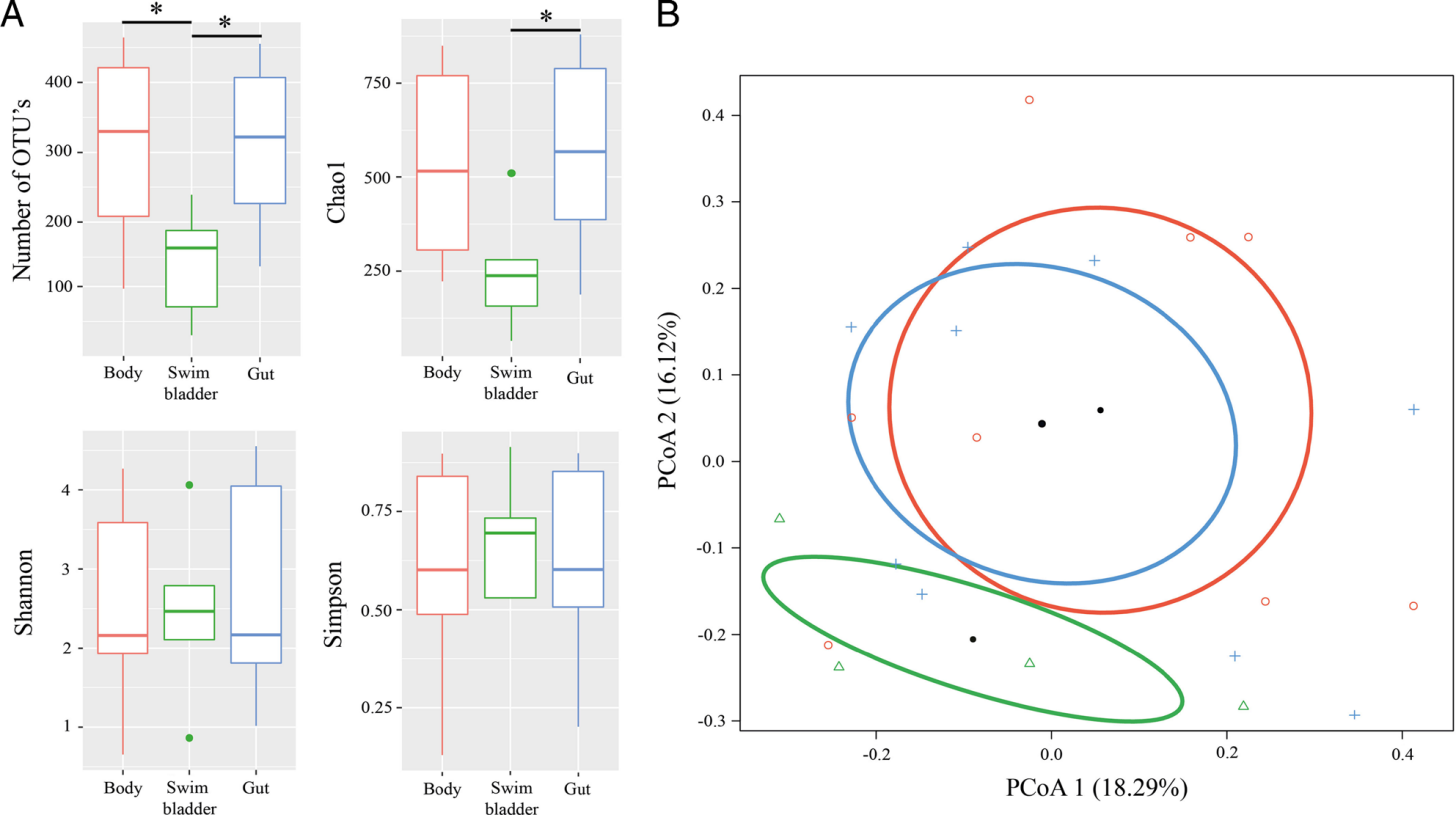
Diversity analysis of microbial community associated with nematodes *Cystidicola farionis*. (A) The richness and diversity estimates of microbial communities. (B) Principal coordinates analysis (PCoA) for nematode-associated microbiota: red—“body”, green—swim bladder, blue—gut. The asterisk character indicates significance at *p* ≤ 0.05.

According to Dunn’s post hoc test the number of observed OTU’s and Chao1 value in the microbial community were significantly (OTU’s: *z* = ‒2.2, *p* = 0.045; Chao1: *z* = ‒2.2, *p* = 0.042) higher in intestine (312.0 ± 41.7 and 567.0 ± 89.8, correspondingly) than in the microbiota associated with swim bladder (167.3 ± 33.4 and 296.4 ± 75.6, correspondingly). Significant differences were also observed in the number of OTU’s between the “body” of nematodes (310.5 ± 46.8) and swim bladder (*Z* = 2.1, *p* = 0.025). No significant differences in richness (number of observed OTU’s and Chao1) and diversity estimates (Shannon and Simpson) were found between microbiota associated with gut and “body” of nematodes (*p* > 0.05). Also, there were no significant differences in Shannon and Simpson estimates found in associated microbiota of nematodes in comparison with the swim bladder of nosed charr (*p* > 0.05) (Fig. 3A).

#### Between different fish morphs

**Figure 4:**
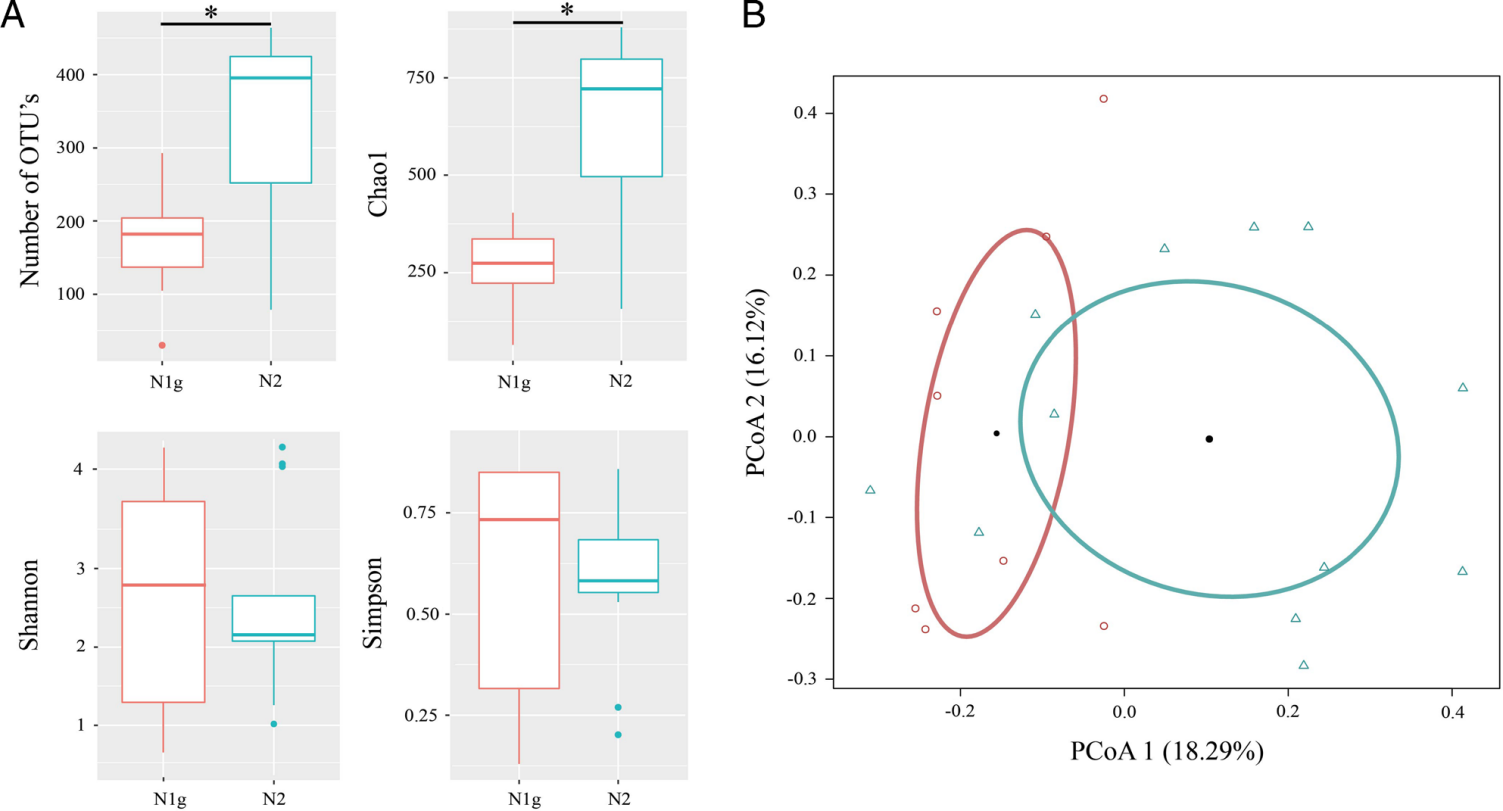
Diversity analysis of the microbial community associated with nematodes *Cystidicola farionis* parasitizing the swim bladder of two morphs of nosed charr. (A) The richness and diversity estimates of microbial communities. (B) Principal coordinates analysis (PCoA) for nematode-associated microbiota: red—morph N1g of nosed charr, blue—morph N2 of nosed charr. The asterisk character indicates significance at *p* ≤ 0.05.

The estimation of the number of OTU’s and Chao1 value of associated microbiota of nematodes and swim bladder has shown with significance, that the highest richness (OTU’s: χ^2^ = 8.9, *p* = 0.003; Chao1: χ^2^ = 9.3, *p* = 0.002) was observed in the nematodes of nosed charr from the morph N2 (345.3 ± 34.6 and 638.8 ± 66.3, correspondingly), while the lowest was detected in the morph N1g (188.3 ± 20.5 and 288.1 ± 26.1, correspondingly) (Fig. 4A). No significant differences in Shannon and Simpson values were found in the microbial community of nematodes from both morphs of nosed charr (*p* > 0.05).

### Associated microbiota of nematodes

**Figure 5:**
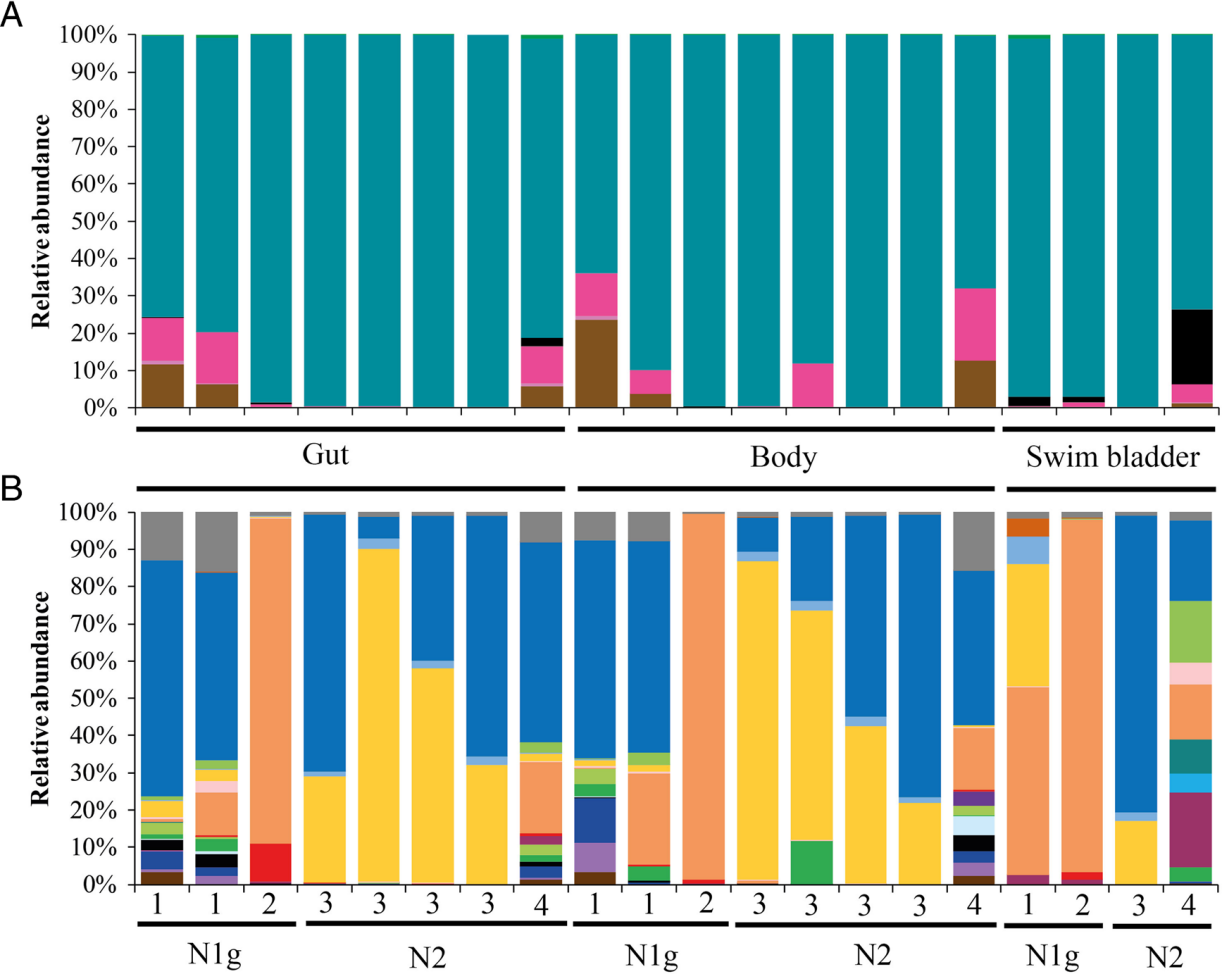
Taxonomical composition of microbial communities identified in gut, “body” remains of *Cystidicola farionis* and microbiota of swim bladder of nosed charr. Numbers 1 to 4 along x-axis denotes the number of individual of nosed charr per pool; N1g/N2 indicates an ecological group of fish, «whole» indicates whole nematode without dissection of their “body”. (A) Phylum composition: (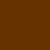) Actinobacteria, (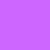) Bacteroidetes, (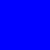) Chloroflexi, (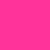) Firmicutes, (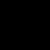) Fusobacteria, (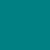) Proteobacteria, (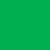), Others<1%. (b) Genus and family proportions: (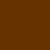) *Corynebacterium* 1, (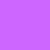) *Lawsonella*, (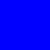) *Cutibacterium*, (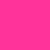) *Nodosilinea* PCC-7104, (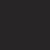) *Tumebacillus*, (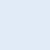) unc. Alicyclobacillaceae, (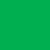) *Bacillus*, (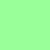) *Staphylococcus*, (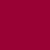) *Cetobacterium*, (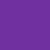) *Caulobacter*, (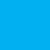) *Paracoccus*, (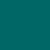) *Pajaroellobacter*, (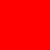) *Aeromonas*, (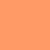) *Shewanella*, (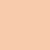) Ambiguous taxa of Enterobacteriaceae, (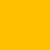) *Yersinia*, (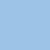) unc. other Enterobacteriaceae, (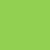) *Acinetobacter*, (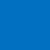) *Pseudomonas*, (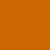) unc. Gammaproteobacteria, (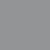) Other < 3%.

Eight, nine and ten phyla were registered in the associated microbiota from swim bladder of nosed charr, and the gut and “body” of nematodes, respectively. In all samples the dominant phyla were Proteobacteria for which abundances ranged from 67.9 to 99.7%. The phyla Actinobacteria, Firmicutes and Fusobacteria were also found in a significant amount (13.7, 19.3 and 20.2%, correspondingly) of the associated microbial communities, but their relative abundances were dependent on the individual hosts. Thus, Actinobacteria and Firmicutes of both intestine and “body” were more abundant in the associated microbiota of individuals 2 and 3 in comparison with the other fish. Fusobacteria were more abundant in the associated microbiota of swim bladder than in microbiota of the gut and “body” of nematodes (Fig. 5A). At the lowest taxonomical level examined, the microbiota of gut and “body” of nematodes was mainly represented by *Aeromonas*, *Pseudomonas*, *Shewanella*, and *Yersinia*. The dominant microbiota of the swim bladder were *Acinetobacter*, *Cetobacterium*, *Pseudomonas*, *Shewanella*, *Yersinia* and unclassified Enterobacteriaceae (Fig. 5B). The relative abundances of the main dominant (with abundance more than 3%) of nematode microbiota also varied between individuals of fish. Thus, *Cutibacterium*, *Lawsonella*, *Staphylococcus*, and *Tumebacillus* were more abundant for both intestine and “body” of nematodes in individuals 2 and 3 in comparison with other fish. The differences in the bacterial community of nematodes parasitizing the swim bladder of nosed charr also depends on the type of tissue analyzed. Thus, *Paracoccus* were registered only in the microbiota associated with the swim bladder, and bacteria from the genera *Lawsonella*, *Staphylococcus*, *Tumebacillus* and unclassified Alicyclobacillaceae were detected in the microbiota of the intestine and “body” of nematodes, in comparison with microbiota of swim bladder.

According to the ADONIS test (data not shown) the microbiota associated with the gut of nematodes was not significantly different from microbial communities of their “body” or swim bladder (p > 0.05), nor between different morphs of fish (p = 0.057). But, the principal coordinates analysis (PCoA) also showed a clear grouping of gut and “body” of nematodes in comparison with microbiota associated with the swim bladder of fish, as well as microbiota of nematodes from morph N1g and N2 (Figs. 3 and 4B). The lack of significance of these observations may be due to the strong inter-individual variation in microbiota composition.

## Discussion

To date, there have been only a few studies that focused upon the bacterial communities associated with fish parasites. Most previous studies have focused on the morphological and physiological characteristics of bacteria associated with parasites inhabiting the fish intestine (Hughes-Stamm et al., 1999; Poddubnaya and Izvekova, 2005; Korneva and Plotnikov, 2006, 2012; Llewellyn et al., 2017; Gonçalves et al., 2020; Kashinskaya et al., 2020, 2021). For example, using the methods of transmission and scanning electron microscopy for examining bacterial diversity, Korneva and Plotnikov (2006) demonstrated that the intestine of *Esox lucius* and the tegument of *Triaenophorus nodulosus* were inhabited not only by microbiota of well-known morphs of bacteria such as various cocci, bacteria from the *Vibrio* group, and rod-like bacteria, but they also documented different morphological forms of very small bacteria with 0.25 to 0.3 nm in diameter and spirochaetes (Korneva and Plotnikov, 2006). Other studies, applying culture-dependent methods, have focused on determination of the gut bacterial diversity of fish in different host-parasite system such as pike *Esox lucius* (*Triaenophorus nodulosus*), stone loach *Barbatula barbatula* (*Proteocephalus torulosus*), perch *Perca fluviatilis* (*Proteocephalus percae*), Eurasian ruffe *Gymnocephalus cernuus* (*Proteocephalus cernuae*). The dominant microbiota identified from those fish was represented by taxa that included opportunistic pathogens from the genera *Aeromonas*, *Vibrio*, *Pseudomonas*, *Shewanella*, *Hafnia*, *Yersinia*, and *Carnobacterium* (Korneva and Plotnikov, 2012). Several authors have also discussed that the symbiotic microbiota from helminths and bacteria from fish intestine may produce different digestive enzymes, such as proteases, that participate in the digestive processes of both the parasite and their host (Izvekova, 2003).

In the present study, the microbiota associated with the intestinal tract and “body” of *C. farionis* as well as microbiota of a swim bladder of the nosed charr were investigated for the first time, using the approach of sequencing the V3-V4 region of the 16S rRNA. As shown early, the nematodes parasitizing the swim bladder of fish may cause several pathological changes of the swim bladder through physical movements of parasites and excretion of toxic metabolites (Menconi et al., 2019). Thus it has been shown, during the swim bladder infestation by *Anguillicola crassus* the migration process of *A. crassus* larval was characterized by epithelial hyperplasia and hyperaemia of the swim bladder wall (Molnár et al., 2016). Despite a higher level of *C. farionis* infestation, parasitological examinations have revealed no significant pathological changes of the swim bladder during the sampling period. In 2014 in Lake Kronotskoe the level of prevalence and intensity of *C. farionis* infestation was studied during the entire period of vegetation (from June to November). It is interesting that during all the months of study the level of prevalence and intensity remained high. Such patterns can be apparently related with selective feeding by the nosed charr on gammarids, which are infested by the invasive stages of *C. farionis* (Busarova et al., 2016). Sokolov and Gordeev have shown that the prevalence and intensity of amphipod infestations by the larvae of these helminths was quite high and reached 21.6% and 0.29, correspondingly (Sokolov and Gordeev, 2014; Busarova et al., 2016). Both morphs of nosed charr (N1g and N2) predominantly feed on gammarids and are characterized by a similar high infestation with *C. farionis* (Esin and Markevich, 2017). As shown early by isotope analysis, these morphs were differentiated into peculiar trophic niches (Esin et al., 2020). Based on these observations, we hypothesized that the associated microbiota of nematodes of both morphs would be different. Our observations are in agreement with their previous findings. According to sequencing results the richness estimates of the microbial community associated with the nematode parasitizing the nosed charr of the N2 morph were significantly higher than the microbiota of nematodes parasitizing the N1g morph of the nosed charr.

The analysis of nucleotide sequences supported that the nematodes studied belong to the species *C. farionis*. We may note that *C. farionis* has low variability based on parts of both the 18S and 28S genes; although identification is supported by the presence of identical sequences (866 b.p. of 28S) in Switzerland from *Salmo trutta*, Italy (756 b.p. of 18S) from a hybrid of *S. trutta* x *S. marmorata*, and USA from *Coregonus clupeaformis* (756 b.p. of 18S).

There is no available information related to microbiota of fish nematodes. During experimental *Pseudocapillaria tomentosa* (Nematoda) infestation the changes of the gut microbiome of zebrafish was only discussed (Gaulke et al., 2016). In comparison to fish, the microbiome research associated with nematodes isolated from different environments has become an increasingly popular area of study. Using the 16S rRNA gene sequencing approaches the microbiota of *Caenorhabditis elegans* (Berg et al., 2016; Dirksen et al., 2016; Samuel et al., 2016), various marine nematodes (Schuelke et al., 2018; Bellec et al., 2019), soil-associated nematodes (Baquiran et al., 2013; Berg et al., 2016; Zheng et al., 2020), a ruminant parasite (Cortés et al., 2020), and plant parasitic nematodes (Yergaliyev et al., 2020) were investigated. To date, the most intensively studied model species for the microbiome research is the nematode *C. elegans* (Berg et al., 2016; Dirksen et al., 2016; Samuel et al., 2016, and others). The functional role of microbiota associated with a natural isolate of *C. elegans* was also analyzed (Zimmermann et al., 2020). According to a recent study, the microbiota of *C. elegans* from natural environments were dominated mostly by Enterobacteriaceae, Pseudomonaceae, Xanthomonodaceae, Sphingobacteriaceae, Weeksellaceae, and Flavobacteriaceae (Berg et al., 2016; Dirksen et al., 2016; Samuel et al., 2016). Microbiota associated with a shallow-water marine nematode *Metoncholaimus albidus* were mainly dominated by Gamma Proteobacteria (*Tiothrix* and to *Pseudoalteromonas*) and Campylobacterota (*Arcobacter, Campylobacter, Sulfurospirillum, Sulfurimonas and Sulfurovum*) (Bellec et al., 2019). Schuelke with co-authors (2018) have observed that the microbiota of nematodes from 33 distinct morphological genera from three distinct geographic areas does not correlate with geographical patterns, or host phylogeny and was dominated by Acidobacteria, Actinobacteria, Bacteroidetes, Firmicutes, Fusobacteria, Gemmatimonadetes, Planctomycetes and Proteobacteria. Most of these studies were focused on estimation of microbiota associated with the gut of nematodes. For this purpose, the nematode-associated microbiota of the intestinal tract was often sampled by washing of nematodes in sterile phosphate-buffered saline, PBS (Hogan et al., 2019), washing with sterile water (Dirksen et al., 2016; Schuelke et al., 2018) or subjecting the nematode’s surface to sodium hypochlorite solution (Zheng et al., 2017, 2020). In contrast, the alternative approach such as an inverted micromanipulation microscope system permits to separate the entire gut and “body” of nematodes mechanically (Murakami et al., 2019). In the present study, we used micro surgery instruments, e.g. similar approach was used by Murakami et al. (2019), as a more relevant method to separate the gut of nematodes from of other nematode’s parts for the study of the respective microbial communities. According to our observation, the microbiota of the gut and “body” of *C. farionis* were represented by similar bacterial dominants such as *Aeromonas*, *Pseudomonas*, *Shewanella*, and *Yersinia*; but due to high intra-individual variability the relative abundances of these bacteria were different in each individual pool of samples. Our results are in agreement with another study that showed species-specific microbiomes with a high inter-individual variability in three cryptic species of marine nematode *Litoditis marina* from shallow water (Derycke et al., 2016).

In the present study, we also characterized the associated microbiota of swim bladder of the nosed charr during a *C. farionis* infestation. As discussed previously, this organ has been considered by several authors to be sterile (Apun et al., 1999); however, a recent study using the sequencing of the V4 region of the 16S rRNA gene, has found bacteria such as *Cohnella*, *Lactococcus* and *Mycoplasma* associated with the swim bladder of healthy rainbow trout *Oncorhynchus mykiss* (Villasante et al., 2019). According to our data, the dominant microbial taxa in the swim bladder of nosed char were *Acinetobacter*, *Cetobacterium*, *Pajaroellobacter*, *Paracoccus*, *Pseudomonas*, *Shewanella*, and *Yersinia*. To date, the method of formation of the bacterial community associated with the swim bladder has not yet been elucidated. It is known that in physostomous fishes, there is a direct connection between the swim bladder and esophagus via the pneumatic duct. This pneumatic duct appears during the late embryonic stage and persists throughout the entire life cycle, while physoclistous fishes lose this connection after the first inflation of the swim bladder (Villasante et al., 2019). Based on these data, a possible access route of bacteria to the swim bladder of healthy fish can be via the pneumatic duct. On the other hand, hypothetically, during parasite infestations the autochthonous microbial communities of the swim bladder can compete with parasite-associated microbiota for ecological niches and nutrient resources, and thereby, changing the biochemical environment of the swim bladder habitat. To confirm or refute of these hypotheses further studies are needed.

In summary, for the first time the microbial community of the nematode gut and “body”, as well as microbiota of the swim bladder of fish were analyzed using a next-generation sequencing approach. Our data has shown that the nematode gut is inhabited by a diverse microbial community which is dominated by *Aeromonas*, *Pseudomonas*, *Shewanella*, and *Yersinia*. High intra-individual variations in the relative abundances of the dominant nematode-associated bacteria were registered. The richness estimates of the nematode-associated microbiota of nosed charr of the N2 morph were significantly higher in comparison with the microbial community of nematodes parasitizing the N1g morph of the nosed charr. Moreover, increasing our knowledge of gut-associated microbiota of fish nematode parasites will help to elucidate more details of microbe-parasite relationships.

## Animal ethics

The following information was supplied relating to ethical approvals (i.e., approving institutional body and any reference numbers): The present research has met the requirements guided by the order of the High and Middle Education Ministry (care for vertebrate animal included in scientific experiments, text number 742 from 13-11-1984) and additionally by the Federal Law of the Russian Federation text number 498 FL (from 19-12-2018) with regard to the humane treatment of animals.
